# Anthropogenic Chromium Emissions in China from 1990 to 2009

**DOI:** 10.1371/journal.pone.0087753

**Published:** 2014-02-05

**Authors:** Hongguang Cheng, Tan Zhou, Qian Li, Lu Lu, Chunye Lin

**Affiliations:** 1 School of Environment, Beijing Normal University, Beijing, China; 2 Spatial Science Laboratory, Texas A&M University, College Station, Texas, United States of America; RMIT University, Australia

## Abstract

An inventory of chromium emission into the atmosphere and water from anthropogenic activities in China was compiled for 1990 through to 2009. We estimate that the total emission of chromium to the atmosphere is about 1.92×10^5^t. Coal and oil combustion were the two leading sources of chromium emission to the atmosphere in China, while the contribution of them showed opposite annual growth trend. In total, nearly 1.34×10^4^t of chromium was discharged to water, mainly from six industrial categories in 20 years. Among them, the metal fabrication industry and the leather tanning sector were the dominant sources of chromium emissions, accounting for approximately 68.0% and 20.0% of the total emissions and representing increases of15.6% and 10.3% annually, respectively. The spatial trends of Cr emissions show significant variation based on emissions from 2005 to 2009. The emission to the atmosphere was heaviest in Hebei, Shandong, Guangdong, Zhejiang and Shanxi, whose annual emissions reached more than 1000t for the high level of coal and oil consumption. In terms of emission to water, the largest contributors were Guangdong, Jiangsu, Shandong and Zhejiang, where most of the leather production and metal manufacturing occur and these four regions accounted for nearly 47.4% of the total emission to water.

## Introduction

Chromium is a naturally occurring metal that is present in small amounts throughout the environment. Human activities have increased the levels of chromium pollution in the air [1], [2]and water [3], [4], [5] and may cause adverse effects on human health and the environment [6], [7], especially in the case of hexavalent chromium Cr(VI) and its compounds.

In China, hexavalent chromium Cr(VI) and its compounds are released into the air as by-products of fossil fuel combustion, waste incineration [8], [9] and various industrial processes (e.g., aerospace products and parts manufacturing, pulp and paper mills, ferrochromium or chromium metal production) [10], [11] as diffused pollution. Meanwhile, chromium is also discharged into the water in the form of wastewater from industries such as leather tanning, metal fabrication and chromium plating [12]. Additional areas of application of chromium include wood preservatives, production of chrome pigments (e.g., lead chromate) which are used in paints, printing inks and anti-corrosive materials.

The extensive use of chromium in various industrial processes also results in the introduction of the metal into soil or landfills. The chromium in the soil may come from atmospheric deposition, sediment accumulation and the potential leachability of chromium slag. According to previous study [13], there is the possibility that chromium contained in the chromium slag could be leached into the soil. However, the leachability of chromium is very low, and it may take 15 to 20 years to lead to a 10% decrease in the total concentration of chromium in a morhorizon through leaching [14] [15], [16] [15], [16]. In addition, chromium salt production bases are relatively concentrated, and there are only approximately 20 factories in China. The pattern of this kind emission can be seen as point pollution, which is easier to control than emission to the air and water. Some previous studies [1], [17] have also compiled Chinese studies related to chromium pollution in the soil. Therefore, we only offer a brief summary of the sources of chromium discharge to the atmosphere and water in this paper.

There have been extensive studies regarding the emission inventories of several volatile trace elements, such as Hg [18], [19], [20], [21], As and Se [22] and Pb [23]. While most of these studies concentrate on the emission from coal combustion or gasoline combustion, a comprehensive investigation of Chromium discharge in China is lacking.

To improve our knowledge and understanding of the status of chromium pollution, a historical perspective can be indispensable when addressing the issues related to long-term heavy metal pollution. Hence, the purpose of this paper is to present a new inventory of emissions of chromium to the atmosphere and water from various sources from 1990 to 2009 and to analyze the temporal and spatial variations of chromium emissions in China. It is anticipated that this study will provide useful information related to the environmental health risks of chromium emissions for policy development in China.

## Methods and Materials

According to previous studies [24], [25], the main sources of chromium emission to the atmosphere are coal combustion, oil combustion, iron and steel production, cement production and waste incineration. In China, the significant sources of chromium discharge to the aquatic system mainly come from industrial wastewater effluent, which includes the leather industry, the chromite mining industry, the chemical manufacturing industry, the non-ferrous smelting industry, the fabricated metal industry and other industries [26], [27].

The methods for estimating emissions include material balances, emission factors and source measurements. Chromium emissions are estimated by using fuel consumption data and detailed chromium emission factors. The basic concept of the chromium emission calculation is described by the following equation:
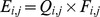
where E is the emission of chromium of one source, Q is the consumption data or industrial production, F is the specific emission factor, i is the sector type, j is the year.

The detailed methods of estimation of chromium for different source can be seen in the first section of Supporting Information where available chromium emission factors are also presented for each source: File S1. Tables–S2 illustrate the average content of chromium in coal as consumed by province and the release rates and control devices of coal combustion; File S1. Tables S10–S11 illustrate the emission factors of different types of oil combustion, ferrochromium production and related industries.

In this study, there are two major categories of all emission sources: atmosphere emission and water effluent emission. The annual consumption data for coal, petroleum products (gasoline, kerosene, diesel, cement production iron and steel production and waste incineration by province are from the China National Bureau of Statistics from 1990–2009. The annual activity level of industries such as the ferrochromium production (File S1. Table S3), the chromites mining (File S1. Table S4) and the leather industry (File S1. Table S5) come from references and online data. In the sectors that emitted chromium to water, such as the fabricated metal industry, non-ferrous smelting, chemical manufacturing and other industries (File S1. Table S6), the sources of these data come from the China Statistical Yearbook. Their specific emission coefficients can be obtained from the Manual for Coefficients of Pollutant Generation and Discharge in Industrial Pollution Sources in China. These data do not include the special administrative regions of Hong Kong or Macao, and they also exclude Taiwan because the data are kept separately for these regions. The detailed information about sources, emission factors and activity data can be seen in Support information.

## Results and Discussion

### Atmospheric Emission

**Figure 1:**
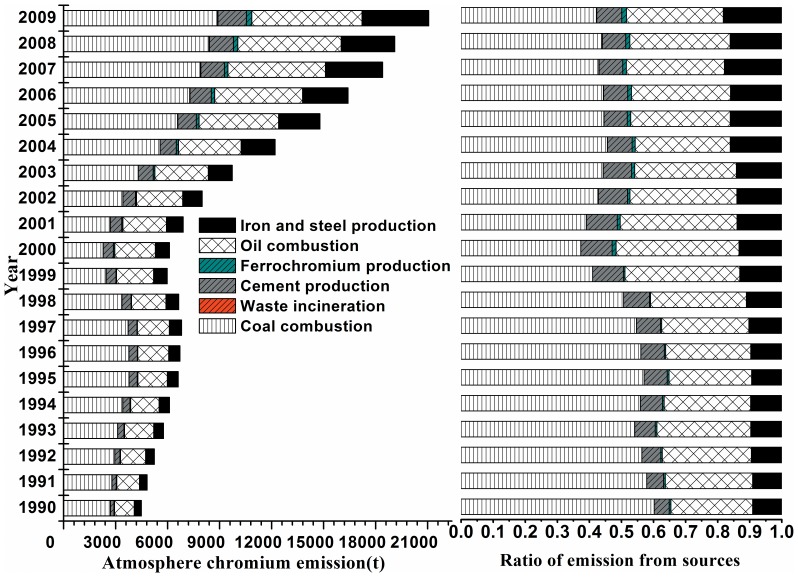
The atmospheric chromium emissions in China from 1990 to 2009.

The total and annual atmospheric chromium emissions from various sources in China from 1990 to 2009 are summarized in Table 1 and Figure 1. The results show that the accumulated total emissions of chromium in China from 1990 to 2009 are estimated to be 1.92×10^5^t. Of the major sources of the total Cr emissions to air shown in Figure 1, coal combustion was the largest emission source, contributing approximately 46.6% of the total. Oil combustion was the second largest source, releasing nearly 5.85×10^4^t of chromium over twenty years, which represented approximately 30.5% of the total chromium emission, followed by the iron and steel industry, the cement industry and ferrochromium production.

**Table 1:** The atmospheric chromium emissions from various sources in China from 1990 to2009.

Source	Emissions (t)	Ratio
**Coal combustion**	8.93×10^4^	46.6%
Power plants	1.56×10^4^	8.1%
Industrial sector	7.37×10^4^	38.4%
Residential sector	4.00×10^1^	0.02%
Other sector	2.60×10^1^	0.01%
**Oil combustion**	5.85×10^4^	30.5%
Diesel	4.05×10^4^	21.1%
Kerosene	4.50×10^3^	2.4%
Gasoline	1.34×10^4^	7.0%
**Ferrochromium production**	1.81×10^3^	0.9%
**Iron and steel industry**	2.75×10^4^	14.3%
**Cement production**	1.46×10^4^	7.6%
**Waste incineration**	8.50×10^1^	0.04%
**Total**	1.92×10^5^	100.0%

#### Temporal trend of chromium emissions by sector

The total emission of chromium from 1990 to 2009 gradually increased, except in 1998. The reduced economic growth caused by the Asian financial crisis [28] in 1998 may be the primary reason for this phenomenon because the chromium-related production activities decreased significantly in this year. This finding also coincides with the inventories of other trace metals, such as Hg [29], Pb [23], Cd and Cr from coal combustion [8]. Generally, the national emission showed a peak value in 1996. Since 2001, the rapid growth of the economy and the increased energy consumption contributed to significant emission increases.

**Table 2:** The chromium discharge into water from various sources in China from 1990 to 2009.

Sources	Emission(t)	Ratio
Nonferrous Metals Smelting and Pressing industry	8.00	0.1%
Fabricated metal industry	9.10×10^3^	68.0%
Leather industry	2.68×10^3^	20.0%
Chemical manufacturing industry	9.90×10^1^	0.7%
Nonferrous Metals Mining and Dressing industry	1.02×10^3^	7.6%
Other Industries	4.82×10^2^	3.6%
Total	1.34×10^4^	100.0%

Coal is the primary energy source in China, and coal combustion has increased dramatically during the past two decades [30]. As is shown in the Figure 1, chromium emissions from coal combustion increased from 2.70×10^3^ t in 1990 to 8.88×10^3^ t in 2009 at an annual average rate of 7.3%. Chromium emissions from coal combustion declined from 1998 to 2000, and the probable explanation is the economic decline that occurred during this period, which is also consistent with the study of Tian [8]. The detailed information about coal combustion and oil combustion by sub-sector are illustrated in File S1. Figures S1–S2.

The annual emission from oil combustion is continuously increasing, which is consistent with the increased demand from civilian-owned motor vehicles [31]. For oil combustion, most of emission comes from diesel and gasoline combustion which account for 69.2% and 23.0% of oil combustion. These two categories comprise the majority of China’s Cr emissions from oil combustion, and their shares kept relatively constant during the past two decades. The emission of kerosene is relative for which the demand and consumption of kerosene is lower than other types of oil. The detailed information about oil combustion can be seen in the Supplementary Information.

Ferrochromium production emitted approximately 1.81×10^3^t of chromium during the period 1990–2009. It is noteworthy that the emissions presented some fluctuations in the first 10 years and continuously increased in the following years. The overall general trend is characterized by growth at 18.1% each year. There were no available data about ferrochromium production from 1990 to 1999, and the estimate seems smaller compare with the measured data in the second ten years. Additionally, the production of other chromium-related alloys, such as nickel chrome, will also emit chromium into the air. In the present study, there were no accurate national data about these alloys. All these factors could lead to an underestimation of chromium emission from ferrochromium production.

It is important to note that the emissions of chromium from the iron and steel industry increased until 2008, which may be due to factors related to the 2008 Olympic Games in China and the global financial crisis of 2008. The ratio of chromium emission from other sources, such as cement production and waste incineration, also increased during this period to a different extent. According to some studies [22], [32], with the development of dust emission requirements and advancement of technology, electrostatic precipitators(ESTs) were gradually substituted by fabric filters (FFs), which are used in cement kilns in China. However, FFs are still not widely used, along with the continuously rapid growth of economic activity and cement consumption. These factors may both contribute to the yearly increase in the emission from cement production in China.

As shown in Figure 1, the emission from iron and steel production is increasing with the high rate of growth at an annual average rate of 12.9%, especially for the recent years. The production and consumption of iron and steel has substantially increased since the 1990s. It has become the world’s largest production base at the beginning of the 21st century, accounting for nearly half of the world’s iron and steel production. Thus, the substantial production of iron may the main reason for the high emission and growth.

As waste incineration is gradually developing, the emissions are also increasing. Although the emission from this sector has been relatively small in recent years, it may have a significant impact on the total emission in the future because municipal solid waste (MSW) has recently been recognized as a renewable source of energy, and waste incineration is playing an increasingly important role in MSW management in China [33]. The detailed information about six sectors’ emission to air is summarized in File S1. Table S7.

#### Spatial variation of chromium emission to air

**Figure 2:**
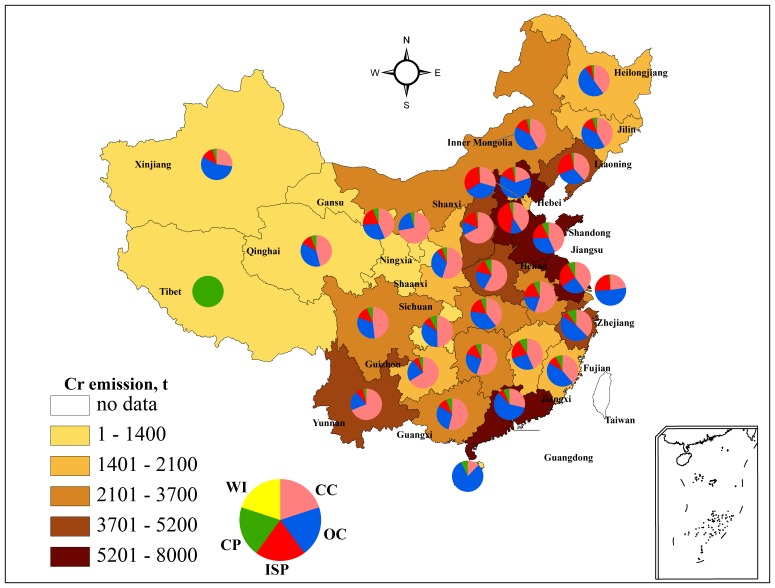
Chromium emissions to atmosphere by region from different sources in China in 2005 to 2009.

The emission of chromium to the air demonstrates significant differences at the province level as is shown in Figure 2. The contributions to the five-year emissions from the top four emission-producing provinces were as follows: 7.95×10^3^t from Hebei, 7.71×10^3^ t from Shandong, 5.28×10^3^ t from Jiangsu and 5.28×10^3^t from Guangdong. The four largest emission regions produced nearly one-third of the total emissions. These regions are located in the east and are traditional industrial bases and economically intensive areas in China. In contrast, Tibet (6t), Qinghai (262 t), Hainan (317 t), Ningxia (644 t) and Gansu (911 t) are the regions with the lowest emissions. Further, notable unevenness can be seen among the provincial inventories. Emissions from the eastern and central provinces are much higher than those in the western regions, except for Yunnan province, in which coal combustion emissions are quite high, corresponding to the rapid increase in the demand for coal-based industries, such as power plants, metallurgy and chemical industries in recent years [34]. In short, the low emission areas are mainly concentrated in areas that are less populated and that have lower economic strength (GDP), i.e., the Northwestern area. The pie chart in Figure 2 represents the emission structure in each region. In most regions, coal combustion or oil combustion contributed more than half of the total emissions. Specifically, chromium emissions from coal consumption are the major cause of pollution in the western area as well as in central China, representing over 50% of the total emissions of the region. Emission from oil combustion occurs in the eastern and southern parts of China, which are more developed and richer regions.

Generally, the majority of the provinces show a peak value of chromium emissions in 2007, followed by a subsequent decline in 2008. It is noteworthy that a negative growth rate appeared in 19 provinces in 2008, including Beijing, Hunan, Jilin, Guizhou and other regions. These findings may imply that the 2008 Olympic Games in China and the global financial crisis in 2008 are the factors lead to the emission decline.

However, the general trend among the provinces shows a high rate of growth. The fastest growth occurred in Qinghai province in 2007 (Tibet is excluded due to a lack of data), with an annual rate of increase of 38.2%, which coincides with the results for lead emission reported by Li [23]. For four municipalities, emissions from Shanghai were nearly twice those of Beijing, Tianjin and Chongqing which is also consistent with Hao’s study of NO_X_ in China [28]. The population, GDP, and gross industrial output value of Shanghai are the highest of these four regions, which may explain this result.

### Discharge into Water

Chromium is a common pollutant introduced into natural waters mainly due to the discharge of wastewater from chromite mining, leather tanning and stainless steel production and other related chromium industries, such as wood preservation, pigment production and electronics manufacturing [1], [14], [35], [36]. Based on national environmental statistical data and the specific chromium emission factors of different sectors, the national gross chromium emission to the water from 1990–2009 was approximately 1.34×10^4^t and detail information about the discharge of chromium to the aquatic environment were summarized in File S1. Table S9.

#### Inventories of chromium emissions by sector

**Figure 3:**
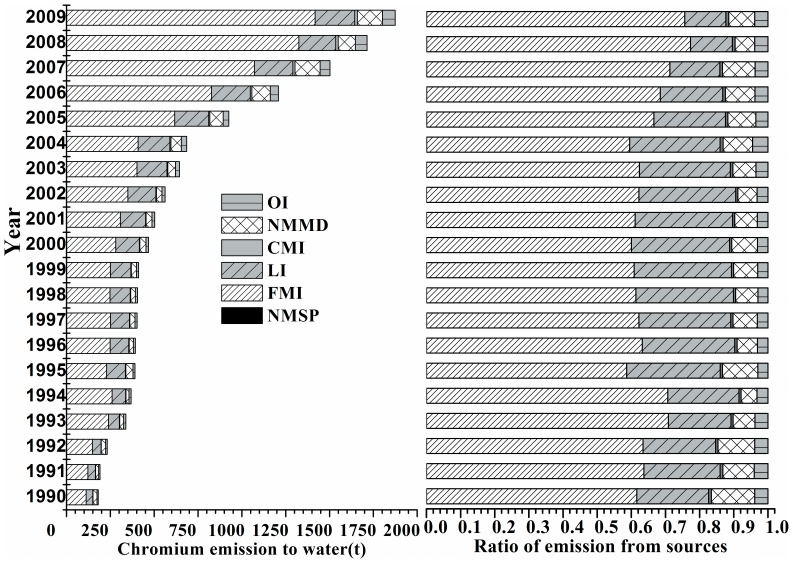
The chromium discharge into the water in China from 1990 to 2009.

There is a general trend for the annual emissions (Figure 3) demonstrating slow growth before the 21th century and a fast increase in recent years. This observation can be partly explained by the rapid growth of industry activities mixed with the management of wastewater discharge by the government [37]. Importantly, there was no general trend for growth in 1997–1999 for any of the sectors, and negative growth was observed in the fabricated metal industry, the nonferrous mining industry and the chemical manufacturing industry despite continuous robust GDP growth of 8.8% in 1997 and 7.8% in 1998. Contrary to earlier expectations, the years of 1997 and 1998 witnessed a decline in China’s output and the reduction of fabricated metal production and other industries, mostly driven by the financial crisis of Asia.

The trend was continuously decreasing over 20 years based on the statistics data from the China Environment Statistical Yearbook. A strict set of standards of wastewater discharge was assumed to be the major reason for this decrease. However, compared with the result of First National Census of Pollution Sources [26]and previous research [26], [38], approximately 2057t heavy metals, including Cd, Cr, As, Pb and Hg, were discharged into the water in 2007. Among them, chromium was the largest contributor with 1643t, which is comparable to the 1502t of chromium emissions of 2007 in the present study.

Of the major source contributions to the total Cr emissions to the water shown in Table 2, the fabricated metal industry contributed over 68.0% of the total emissions, and the leather industry contributed 20.0%. These two sectors comprise over 88.0% of total chromium emissions to the water in China. The share contributed by the fabricated metal industry remained relatively constant in the past decades, while it was the largest chromium emission contributor during this period. Chromium emissions to water from this sector increased from 111t in 1990 to 1.42×10^3^t in 2009 with an annual growth rate of 15.6% in 20 years. From 2005 to 2009, the annual growth rate reached more than 29.1%, and this may be attributed to the drastic development of the automobile industry, the construction of highways and railways and the related industries in these years during which China’s GDP doubled [39].

The contribution of the leather industry experienced a gradual increase followed by a continuous decline in recent years, accounting for 12.0% of the total chromium emissions in 2009. The emissions increased from 38t in 1990 to 226t in 2009, and the average annual growth rate was 10.3%. Specifically, the emission from heavy leather production was approximately 1.6×10^3^t, which is higher than the emission from light leather production with 1.08×10^3^t. The low utilization of chromium and relatively backward tanning process [40], [41], [42]may be the primary reasons for the increased emission of chromium from the leather tanning industry. It is reported that only 60%–70% of the chromium is used in the tanning process, and 10% of the residual liquids are directly discharged into the river [40]. Interestingly, there was a decline in 1997–1998 for nonferrous mining. The nonferrous mining and dressing industry pressing industry was the next largest contributor, and the emissions increased rapidly in recent years, from 76t in 2005 to 143t in 2009, with an approximately 23.5% annual growth rate.

Particular attention should also be paid to other industries, such as manufacturing of textile, electronic equipment and furniture etc., which released 482t chromium during the period 1990 through 2009and account for 7.6% of the total emissions. With the recently attention and intensity of the government’s control of heavy metal pollution in China, the emission of chromium from different sources has been gradually reduced. For the non-ferrous smelting and chemical manufacturing industries, the annual emissions were relatively stable in the beginning and gradually increased in recent years, accounting for less than 1.0% altogether. Although the emission from these sources is small, the concentration of emissions in the absence of effective control equipment and standard process represents a significant health hazard for the surrounding population [23]. Additionally, the rapid economic growth mixed with the over standard discharge of wastewater in local areas [43] will present a challenge to the prospect of reduction.

Therefore, to achieve reductions in chromium emissions into the water, China should mainly aim to eliminate emissions from the fabricated metal industry and leather tanning by employing advanced process and treatment technologies. Additionally, the government should enforce standards of wastewater emission to reduce local emissions.

#### Spatial variations in the emission of chromium to water

The national annual emission of chromium was combined with the ratio for each specific province to yield estimations of the emissions at the provincial level (Table 3). From 2005 to 2009, the emissions of the provinces showed a peak value in 2007, followed by a decline in 2008. The trend is consistent with the national and provincial emission to air from 2005 to 2009. On the whole, the emissions of chromium are highly concentrated in the provinces of the eastern, central and southern regions, such as Guangdong, Zhejiang, Jiangsu, Hubei and Hunan. The chromium emissions of the different provinces in present study are very similar to the results of Wu (2012), who also singled out eight provinces, including Guangdong, Zhejiang, Jiangsu and Hunan, as preferential control areas for heavy metal pollution from wastewater.

**Table 3:** Summary of Total Chromium Emission Estimates (t) to water by Province from 2005 to 2009.

Region	2005	2006	2007	2008	2009	Total
**Northern Region**	**120**	**154**	**187**	**219**	**233**	**912**
Beijing	25	31	36	35	38	165
Tianjin	25	33	37	42	45	182
Hebei	42	51	63	78	82	317
Shanxi	17	23	29	34	32	134
Inner Mongolia	10	16	22	30	37	113
**Northeastern Region**	**69**	**93**	**115**	**138**	**155**	**570**
Liaoning	38	54	68	84	96	340
Jilin	15	18	24	28	34	120
Heilongjiang	16	21	23	26	25	111
**Eastern Region**	**435**	**565**	**698**	**782**	**852**	**3332**
Shanghai	61	71	83	85	82	381
Jiangsu	123	158	198	229	250	958
Zhejiang	88	111	134	138	140	611
Anhui	18	23	29	38	46	153
Fujian	31	38	46	51	57	225
Jiangxi	11	16	23	29	33	113
Shandong	103	148	185	213	243	892
**Central and Southern**	**221**	**291**	**368**	**418**	**455**	**1752**
Henan	38	53	76	88	95	350
Hubei	22	28	36	45	53	185
Hunan	18	23	31	39	46	158
Guangdong	131	170	205	221	233	961
Guangxi	9	13	17	21	24	83
Hainan	2	2	4	4	4	15
**Southwestern Region**	**49**	**64**	**83**	**97**	**115**	**407**
Chongqing	11	12	16	19	23	82
Sichuan	22	30	41	50	62	205
Guizhou	6	8	9	11	12	46
Yunnan	10	13	16	17	18	74
Tibet	0	0	0	0	0	1
**Northwestern Region**	**31**	**42**	**52**	**60**	**64**	**251**
Shaanxi	13	17	21	25	29	105
Gansu	7	9	12	12	13	54
Qinghai	2	2	3	4	4	15
Ningxia	3	3	4	5	5	19
Xinjiang	7	10	12	14	14	57

The heavy emissions from these areas are driven by high levels of industrial activities and large population. Guangdong is the largest producer of fabricated metal, Hunan is rich with mining activities, and most of the leather tanning industries are located in Zhejiang, according to survey data [26]. On the contrary, most of provinces in the western part of China had low chromium emissions into the water, except Shaanxi and Sichuan. This may be mainly due to the increasing labor cost in the developed eastern coastal areas. Motivated by the policies of the western local governments, some highly polluted manufacturing enterprises are moving to these provinces.

Interestingly, the chromium emissions in Shaanxi in2008 differed from the normal levels, which reached suddenly85t. It is difficult to identify the exact reason for this result because we have no detailed data for this province. The relative backward water pollution controlling facilities that lead to ineffectively treated industrial wastewater and urban sewage being directly discharged into the rivers may have contributed to this [44]. Consequently, attention should also be concentrated on the emissions of western regions due to the potentially harmful impacts of emission on the local environment and human health.

### Release of Chromium to Ecosystems

Estimations of the chromium emissions to the atmosphere and water from1990 to 2009 are presented in Table 4. To better predict the trend, the two decades are divided into four periods, which could provide a more typical reflection of the emission status.

**Table 4:** Summary of chromium emission to atmosphere and water in different periods (t).

Category	Sources	1990–1994	1995–1999	2000–2004	2005–2009	sum
To air	Coal combustion	1.50×10^4^	1.71×10^4^	1.83×10^4^	3.90×10^4^	8.93×10^4^
	Waste incineration	0.00	0.00	9.01	7.65×10^1^	8.55×10^1^
	Cement production	1.56×10^3^	2.59×10^3^	3.78×10^3^	6.70×10^3^	1.46×10^4^
	Ferrochromium production	1.40×10^2^	1.40×10^2^	4.18×10^2^	1.11×10^3^	1.81×10^3^
	Oil combustion	7.24×10^3^	9.45×10^3^	1.42×10^4^	2.76×10^4^	5.85×10^4^
	Iron and steel production	2.50×10^3^	3.50×10^3^	6.25×10^3^	1.52×10^4^	2.75×10^4^
	Total per year	5.28×10^3^	6.55×10^3^	8.59×10^3^	1.80×10^4^	3.84×10^4^
To water	Nonferrous smelting and Pressing	0.52	0.68	1.20	5.25	7.65
	Fabricated metal industry	8.79×10^2^	1.22×10^3^	1.74×10^3^	5.25×10^3^	9.10×10^3^
	Leather industry	2.68×10^2^	5.54×10^2^	7.90×10^2^	1.07×10^3^	2.68×10^3^
	Chemical manufacturing industry	9.00	1.30×10^1^	2.02×10^1^	5.70×10^1^	9.92×10^1^
	Nonferrous Mining and Dressing	1.04×10^2^	1.48×10^2^	2.03×10^2^	5.65×10^2^	1.02×10^3^
	Other industries	4.83×10^1^	5.99×10^1^	1.01×10^2^	2.73×10^2^	4.82×10^2^
	Total per year	2.62×10^2^	4.00×10^2^	5.72×10^2^	1.44×10^3^	2.68×10^3^

Emissions into the air increased during these four periods, and the most rapid increased occurred from 2005 to 2009, and rate of growth over these five years (there were no available data about waste incineration before 2003) reached over 100.0%, which coincides with the economy and energy use during this period. China’s GDP increased by a double-digit annual average during the period of 2005 to 2009, reaching as high as 10.2%. Specifically, the most drastic change came from ferrochromium production, and the trend became increasingly obvious at the beginning of the 21st century because the majority of ferrochromium is used in the stainless steel industry, reaching 85% [45]. The increasing demand for stainless steel may be the major reason for this dramatic change. In contrast, coal combustion presented the lowest growth rate, while it remains the largest contributor to emissions.

For emissions into water, all sectors experienced gradual growth in the first three periods and drastic increases in the last period. This increase from 2005 to 2009 is also consistent with the industrial production and rapid development in recent years. The drastic change is associated with the fabricated metal industry, which nearly tripled its emission in the third period (2000–2004). For the non-ferrous smelting industry and the chemical manufacturing industry, the growth rates were much smaller. Based on the China Statically Yearbook from 2000–2009, the development of these sectors has been less obvious. Additionally, the attention to environmental protection and the implementation of discharge standards has contributed to this smaller growth [46], [47].

It is also noteworthy that emissions into the air were much larger than emissions into the water. However, it is not correct to say that the emission of chromium pollution into the atmosphere is more severe than the emission into water because the emission of chromium into the air is dissipated over a large area, while chromium discharge to water flows along rivers, and which has a line distribution, making it much easier for chromium contamination to enter the food chain and have a more direct effect on human life and health.

### Speciation of Cr Compounds

The speciation of Cr compounds for each emission source sector is pivotal to accurately assess the environmental and human health impacts since toxicity of Cr(III) (non-carcinogenic) and Cr(VI) (carcinogenic) to organism varies considerably. There are a large amount of chromium with +2, +3, +6 or in combination are introduced into environment. It is well established that chromium is mobilized in association with airborne particles derived from high-temperature combustion sources, like coal, oil combustion and waste incineration. For chromium in industrial wastes, cement-producing, it predominantly occurs as the hexavalent form in chromate (CrO_4_^2−^) and dichromate (Cr_2_O_7_^2−^) ions. Emissions of chromium from the processing of raw material like smelting or mining, chromium plating would predominantly be in the trivalent oxidation state. However, the limited amounts of data related to chromium speciation of each source make the detailed emissions estimation unavailable.

## Uncertainty Analysis

Because the emission of chromium comes from so many sources and most of the activity data indirectly come from references or statistical reports, an uncertainty analysis is necessary to better understand the estimation of the emissions.

In general, there are three aspects of the uncertainty of the emissions that need to be considered: commercial energy use, emission factors and emission control [48]. In the present study, these three factors apply to atmosphere emission, while only the first two contribute to wastewater emissions because emission control data are incorporated into the discharge coefficient in the Manual for Coefficients of Pollutant Generation and Discharge in Industrial Pollution Sources.

For atmosphere emission, the commercial energy use data come from China’s official statistics yearbook, and the figures are somewhat underestimated according to some studies [18], [19]. There are two reasons for this underestimation: one is that the data may have been adjusted for political reason and using data from a multitude of sources could reduce the uncertainty of the output. Another reason is that many of the activities that release large amounts of chromium occur in remote parts of the country (and may actually be illegal). Residential coal use in rural areas may be under-reported in Chinese statistics.

With regard to the emission factors, most of them were cited from EPA and EEA because there are no data available in China. The uncertainty may come from differences in boiler combustion efficiency and process technologies used in China. However, information about the chromium contents of raw materials by province can improve the accuracy of the estimation. The control devices used by combustion industries are also diverse across different provinces, and most industries installed advanced devices after entering 21th century. Therefore, to adopt the average values of the measurements from foreign and domestic studies to balance this change could make it more reasonable. There is less detailed information about the activities of industries that discharge chromium into the water, and the statistical data on chromium emissions into the water were indirectly obtained though the industrial output in China Statistical Yearbook. While the emission coefficients are choose from China’s research which may enhance the accuracy of estimation. Compared with measured data of First National Census of Pollution Sources, it is suggested that the estimations are located in the reasonable ranges. In addition, the two major sources, namely the leather industry and chromite mining, were recalculated by combing the activity data with the discharge coefficient to reduce the possible underestimation of the emission.

It is inevitable that estimations of emissions will contain uncertainties. Additional detailed investigations and field tests for all chromium-related industries will be helpful for a complete understanding of the emissions of chromium to the environment. Furthermore, there is little information about the emission of chromium, which could provide a basis for policies aiming for emission control in China.

## Conclusion

We present, for the first time, a detailed estimation of chromium emissions to the environment from different sources by province during the period from 1990 to 2009. The national total atmospheric emissions of chromium were estimated at approximately 1.92×10^5^t, at annual growth rates of 8.8% since 1990. Coal combustion was identified as the largest contributor, accounting for 45.6% of the total atmospheric emission. However, the contribution of coal decreased from 60.2% in 1990 to 42.2% in 2009. Conversely, the contribution of oil combustion to chromium emissions has grown slightly during these years, which may be because coal has been replaced with cleaner fuels, such as oil, natural gas and LPG, in recent years. Other sources of emission have also increased at different rates, coinciding with the rapid economic development in China. As for emission into water, the national total emission has undergone fluctuations that may have been caused by a combination of industrial growth and the implementation of wastewater discharge standards. The fabricated metal industry and the leather tanning industry were singled out as the major emission sources, accounting for 68.0% and 20.0% of the total emission to water. Other industries, such as non-ferrous smelting, mining and fabricated metals, were also not negligible sources.

The spatial characteristics of emissions to air and water are based on estimation from 2005 through 2009. Guangdong, Zhejiang, Jiangsu, Shandong and Hebei are the provinces with the largest emissions in China. The emissions are concentrated in the costal and central areas of China, and the regions with severe pollution are slowly moving toward western areas, such as Gansu, Yunnan and Shaanxi. This phenomenon may be due to the policies of the local governments and environmental pressure of the coastal region. The magnitude of these emissions may be debated, but their growing importance is unquestionable. To improve our knowledge and understanding of the current scenario, it is necessary to establish a complete field testing system and pay more attention to the forms of chromium in the environment.

## Supporting Information

Figure S1 **The temporal trend of emission of chromium from coal combustion.** *For four sectors of coal combustion have drastic difference of the industry sector and power plant sector based on the left vertical axis, and others and residential sector based on right vertical axis. Among all of the coal consuming sectors, the chromium emissions from the power sector are increasing the fastest, with an average 7.91% annual increase, reaching 1407 t in 2009. However, the main contributor to coal combustion is the industry sector, which remained nearly constant throughout study period, representing over 80% of coal combustion. Another highlighted feature is that the emissions of chromium from the industry have increased substantially since 2005. The rapid expansion of energy-intensive manufacturing industries, such as steel and cement production, and coal consumption by industrial sector after the recovery of the economic decline may explain this rapid growth. The growth rate of power plants is lower compared with that of the industrial sector, and negative growth was observed in 2004 and 2008. This may due to the improvement of PM and SO_2_ control devices in coal-fired power plants [29]. Emission from the residential sector and other sectors declined at the beginning of the study period and then increased after 2003. This fluctuation may be a co-effect of the substitution of coal with cleaner fuels, such as natural gas, and the gradual increase of coal consumption in recent years.
(TIF)

Figure S2 **The temporal trend of emission of chromium from oil combustion.**
(TIF)

File S1 Table S1. Average contents of Cr in raw coals consumed in China by provinces. Table S2. Release rates and control devices of Cr for coal combustion. Table S3. The estimated emission of ferrochromium production into atmosphere, 1990–2009. Table S4. The estimated discharge into water from nonferrous mining industry. Table S5. The estimated discharge into water from leather tanning industry. Table S6. Industrial output values of sectors and discount rate of China from 1990 to 2009. Table S7. The estimated atmospheric chromium emissions in China, 1990–2009. Table S8. The estimated atmospheric chromium emissions by region in China, 2005–2009. Table S9. The estimated chromium emissions to water in China, 1990–2009. Table S10. Emission factors of different types of oil combustion. Table S11. Emission factors of ferrochromium production and related industries.
(DOCX)
