# Supplementary material for: Anthropogenic Chromium Emissions in China from 1990 to 2009
Source: PLoS One. 2014 Feb 5;9(2):e87753. doi: 10.1371/journal.pone.0087753 (PMC3914863; doi:10.1371/journal.pone.0087753)
Supplement: File S1 — Table S1. Average contents of Cr in raw coals consumed in China by provinces. Table S2. Release rates and control devices of Cr for coal combustion. Table S3. The estimated emission of ferrochromium production into atmosphere, 1990–2009. Table S4. The estimated discharge into water from nonferrous mining industry. Table S5. The estimated discharge into water from leather tanning industry. Table S6. Industrial output values of sectors and discount rate of China from 1990 to 2009. Table S7. The estimated atmospheric chromium emissions in China, 1990–2009. Table S8. The estimated atmospheric chromium emissions by region in China, 2005–2009. Table S9. The estimated chromium emissions to water in China, 1990–2009. Table S10. Emission factors of different types of oil combustion. Table S11. Emission factors of ferrochromium production and related industries. (DOCX) [file pone.0087753.s003.docx]

**Supporting Information**

# Anthropogenic Chromium Emissions in China from 1990 to 2009

Hongguang Cheng ^1*^, Tan Zhou^1,2^, Qian Li^1^, Lu Lu^1^, Chunye Lin^1^

1 School of Environment, Beijing Normal University, Beijing 100875, China

2 Spatial Science Laboratory, Texas A&M University, College Station, TX, USA chg@bnu.edu.cn

# Sources of emission

## Atmosphere emission sources

### Coal combustion

The amount of chromium emitted to the atmosphere during smelting and burning of coal is dependent primarily on the following factors: the chromium content of the coal, the type of boiler used and its firing configuration and the chromium removal efficiency of any controls that may be present. According to the previous study [1] of the inventory of chromium, by incorporating the provincial-level coal consumption data and the detailed emission factors associated with power plants, industry, residential use and other use sectors, the basic formula of chromium can be expressed as follows:

$$E_{i,j}=C_{i,j}\times A_{i,j}\times F_{i,j}\times(1-P)\times(1-P_{FGD})$$

where E is the emissions of atmospheric Cr; C is the averaged chromium content of coal consumed in one province; A is the amount of coal consumption; F is the fraction of Cr released from coal combustion; P and P_FGD_ are the fractions of the chromium removal efficiency of the existing dust collectors (cyclones, wet scrubbers, electrostatic precipitators, fabric filters, etc.) and flue gas desulfurization (FGD) devices, respectively; i is the province (autonomous region or municipality); and j is the emission source classified by economic sector and combustion facility and the equipped particulate matter (PM) and SO_2_ control devices. The average content of chromium in coal as consumed by province and the release rates and control devices of coal combustion are illustrated in File S1. Tables S1 - Table S2.

#### Averaged content of Cr in raw coal consumed

In coal burning areas, the different contents of Cr in the coal have affected the emission of chromium into the atmosphere. Specifying the chromium content of coal for different regions tends to make the results more accurate. In this paper, the content of Cr in different provinces are summarized from the available published literatures[1,2,3,4,5]. The average chromium content in coal from geographically neighboring provinces can be used if no values are available from the existing literature. Most of data come from Tian [1] for it is more reasonable to use consumed coal products of one province which was calculated by a coal flow matrix. The average content of Cr in coal as consumed by province is illustrated in the File S1. Table S1.

#### Removal efficiencies of PM and control equipments

The release efficiency of Cr is different for combustion technologies, operation conditions and sectors. Therefore, it is necessary to consider the average efficiency of boilers, particulate matter (PM) control devices, and FGD devices. The major combustion type for coal-fired power plants in most of the provinces in China is pulverized-coal boilers which account for over 90% of the total, and the remaining share is divided between fluidized-bed furnaces and stoker-fired boilers; while the stoker-fired boiler is the dominant boiler type used in the industrial sectors[1]. Presently, all coal-fired power plants in China have adopted particulate control devices and the share of electrostatic precipitators (ESP) has reached more than 95%. More and more ESP and FFs have also been introduced into the industrial sector in recent years for complying with more stringent emission standards[6]. Since the technologies used in these sector are vary in different periods, we assume that average values of those reported in available references as the average removal efficiencies by different PM control devices. The SO_2_ control devices of power plant were mainly coal washing and wet-FGD before 2000 and wet-FGD was the following years.

For industrial sector, the PM control devices were also mainly cyclone and wet scrubber before 2000 and with the introduction of ESP and FFs in the following years, and its removal efficiency is the average of the four devices. While the industrial sectors do not need to install FGD devices [7], so the FGD was taken into account. The detailed information is shown in the File S1. Table S2.

The commonly-used stoves in the reported literature are applied in residential and other sectors for coal-fired facilities are mainly traditional and uncontrolled small scale stoves without any PM and SO_2_ control devices. However, there is little research on the emission release rate for residential and other coal combustion applications, and for this reason the emission factor of 1.92×10^-5^ was chosen as a reference value[8].

### Oil combustion

Chromium is a trace element that is common in most oils, and some research [2] has concluded that fossil fuel combustion continues to be the major source of Cr. The combustion of oil produced essentially no bottom ash, minimizing the effect of the boiler type and firing configuration on the level of chromium emissions from oil fuels.

Therefore, the emissions from oil combustion can be estimated based on fuel consumption and specific emission factors. In this study, the oil combustion sources are divided into three major categories: gasoline, kerosene and diesel. Limited chromium emission factors are available for the combustion of oil. Based on a study by the EPA and EEA, the emission factors for four categories are shown in File S1. Table S10.

### Ferrochromium production

Ferrochromium plants are also a major source of chromium emission to the air [9,10].Previous studies have shown that most of ferrochromium is used in stainless steel production, and the proportion could reach more than 80% in China. There are no available data about the output of ferrochromium at the national level before 2000, and studies of the emission factor of ferrochromium production are also lacking in China because most ferrochromium use occurs in stainless steel production. To estimate the amounts of chromium emission to the air, we assumed that the production of ferrochromium corresponded to the amount of stainless steel production, where 0.45 t ferrochromium could produce 1t stainless steel [11]. The output of ferrochromium and emission are summarized in File S1. Table S3. Because China has not performed sufficient research on emission factors, we selected the emission factors presented by the US Environmental Protection Agency. The emission factors used in the present study are listed in File S1. Table S11.

### Other sources

The other sources of chromium include cement, iron and steel production and waste incineration. China has become the largest producer of cement, iron and steel [12]. The production of cement, iron and steel each increased significantly in China during the past decade, at an annual growth rate of more than 7.0%.

The primary chromium emission sources in cement production come from kiln operations and grinding mills. In China, shaft kilns represented over 80% of the production capacity in 1990s [13]. While the elimination of shaft kilns and technological advances have resulted in decreasing emissions in recent years, the emission factors obtained during recent years are more appropriate for the actual conditions. In this study, the emission factor for cement production during 1990-2009 was assumed to be 1 g/t [2].

For iron and steel production, the emission factor was assumed to be 2.3 g/t and 4.5 g/t for pig iron production and steel production [2] from 1990 to 2009. We assumed that these emission factor values did not change during the period 1990-2009.

Estimation of emission from the incineration of waste disposal is difficult because of a lack of detailed information on the amounts of waste incinerated before 2003 and the content of chromium in the wastes. Therefore, the emission factors for chromium in Western Europe and the United States were directly used in this article.

These estimates were performed on the basis of emission factors and statistical information on the pig iron and steel production and cement production. The average values of these factors are presented in File S1. Table S11.

## Water effluent emission sources

The release of heavy metals into the environment through industrial effluents has become a major concern during the last decades [14,15], even if the majority of metals are transferred to sewage sludge. For chromium, however, the contribution from domestic wastewater is negligible compared to the discharge of industry wastewater into the water because there is almost no chromium contained in the domestic wastewater. Therefore, the chromium discharged into water mainly came from the following six industries: non-ferrous mining industry, leather tanning industry, fabricated metal industry, non-ferrous smelting industry, chemical manufacturing industry and other industries in the present study.

### Nonferrous mining industry

The emissions of chromite ore occur primarily during mining and ore dressing. Most of the chromite in China depends on export from other countries, such as India, Iran, Pakistan and Vietnam. In recent decades, this type of external dependence has reached 95% for the chromite-poor areas in China. The production of chromite is divided into two parts: import chrome ore and domestically mined chromite ore. The emission co-efficient is affected by many factors, such as mining processes, scale and raw materials. In this article, the emission factor for the two categories is taken from the Manual for Coefficients of Pollutant Generation and Discharge in Industrial Pollution Sources of China. It is assumed to be the average of different processes, 54.3 g/t and 21.5 g/t, respectively. The detailed information about the production of chrome ore is summarized in the File S1. Table S4.

### Leather tanning industry

The chromium emissions to the water resulting from the leather tanning industries could be estimated based on the production of leather and the specific emission factor. The production of leather could be divided into two sections: light leather and heavy leather. The final output of heavy leather is shoe leather, according to the China Market Yearbook. The production of leather could be assumed to represent light leather and shoe leather. The determination of the emission factors depends on the methods of wastewater treatment and material used for the leather. In general, there are three types of technologies used to process the wastewater from the leather industry in China: physical + chemical, chemical + aerobic biological treatment and chemical + a combination of bio-processing. The emission factors of chromium from these processes are 50 g/t, 20 g/t and 20 g/t, respectively. In the present study, we used the average emission co-efficient of these three technologies, 30 g/t, as the final emission co-efficient of chromium emission for different technologies implemented by China for wastewater treatment. The detailed data on the emission is summarized in File S1. Table S5.

### Other sources

The other sources of chromium emission include the fabricated metal industry, non-ferrous smelting, chemical manufacturing and other industries (textile industries, furniture manufacturing, timber processing etc.). There are no exact production data and the emission factors of these sectors. To better predict the emission of these sectors, we use the gross industrial output, which came from the China Statistical Yearbook, and the specific industry emission co-efficient, which was calculated by the measured emission data from the First National Census on Pollution Sources to estimate the amount of chromium into water. Due to the rapid development of China over these 20 years, we considered the discount rate of the gross industrial output value to net present value. The industrial output value of each sector and discount rate are summarized in File S1. Table S6.

Thus, in this study we used the following formula to calculate the emission from fabricated metal industry, non-ferrous smelting, chemical manufacturing and other industries.

$$E_{i}=\frac{G_{i}}{d_{j}}\times F_{i}$$

Where E was the emission of chromium, G is the gross industrial output, d is the discount rate of one year, F was the specific emission co-efficient, i is the sector and j is the year (1990-2009).

# Tables

**Table S1.Average contents of Cr in raw coals consumed in China by provinces (unit: ug/g )**

| Provinces | Cr content | Provinces | Cr content |
| --- | --- | --- | --- |
| Anhui | 29.84 | Jiangxi | 34.44 |
| Beijing | 25.47 | Jilin | 18.35 |
| Chongqing | 28.89 | Liaoning | 23.39 |
| Fujian | 29.78 | Ningxia | 27.12 |
| Gansu | 23.16 | Qinghai | 27.98 |
| Guangdong | 32.65 | Shaanxi | 32.23 |
| Guangxi | 55.38 | Shandong | 21.93 |
| Guizhou | 28.49 | Shanghai | 25.98 |
| Hainan | 28.05 | Shanxi | 21.82 |
| Hebei | 26.55 | Sichuan | 33.12 |
| Heilongjiang | 15.60 | Tianjin | 25.05 |
| Henan | 24.84 | Tibet | 13.07^a^ |
| Hubei | 28.03 | Xinjiang | 13.07 |
| Hunan | 33.73 | Yunnan | 70.79 |
| Inner Mongolia | 14.75 | Zhejiang | 26.80 |
| Jiangsu | 25.92 |  |  |

a The value is assumed to be equal to that for Xinjiang province due to lack of information.

Almost all values come from [1] who used coal flow matrix among 30 provinces in the Chinese mainland to calculate Cr content of coal consumed by province and its confidential interval is 95%.

In coal burning areas, the different contents of Cr in the coal have affected the emission of chromium into the atmosphere. Specifying the chromium content of coal for different regions tends to make the results more accurate. In this paper, the content of Cr in different provinces was summarized from the available published literature [1,2,3,4,5]. The average chromium content in coal from geographically neighboring provinces can be used if no values are available from the existing literature. Most of data come from Tian et al. [1] because it is more reasonable to use the consumed coal products of one province, which was calculated by a coal flow matrix.

**Table S2.Release rates and control devices of Cr for coal combustion**

| Category | Devices | Release rate(%) | Sources |
| --- | --- | --- | --- |
| Release rate (%) | Pulverized-coal boiler | 88 | [16] |
|  |  | 93 | [17] |
|  |  | 84.5 | [1] |
|  | Stoker fired boiler | 28.47 | [18] |
|  |  | 25 | [19] |
|  | Fluidized-bed furnace | 80 | [20] |
|  |  | 82 | [17] |
|  | Coke furnace | 28 | [21] |
|  |  | 20 | [18] |
| Category | Devices | Removal efficiency (%) | Sources |
|  | ESPs | 98 | [22] |
|  |  | 99.7 | [23] |
|  |  | 96.85 | [24] |
|  | FFs | 87 | [16] |
| PM control devices |  | 99.0 | [25] |
|  |  | 99.4 | [24] |
|  | Wet scrubber | 48.14 | [8] |
|  | Cyclone | 17.77 | [18] |
|  |  | 42.30 | [8] |
| SO_2_ control devices | Wet-FGD | 80 | [23] |
|  |  | 92 | [1] |
| In this study |  |  |  |
|  | Release rate (%) | Removal efficiency (%) | |
|  |  | PM control | SO_2_control |
| Power plant | 81.45 | 65.97 | 86 |
| Industry | 45 | 67.96 | / |

**Table S3. The estimated emission of ferrochromium production into atmosphere, 1990-2009**

| Year | Stainless steel production(10^4^t) | Ferrochromium production(10^4^t) | Emission(t) |
| --- | --- | --- | --- |
| 1990 | 24.00 | 12.71 | 24.14 |
| 1991 | 26.00 | 13.76 | 26.15 |
| 1992 | 25.00 | 13.24 | 25.15 |
| 1993 | 31.90 | 16.89 | 32.09 |
| 1994 | 32.50 | 17.21 | 32.69 |
| 1995 | 38.00 | 20.12 | 38.22 |
| 1996 | 27.00 | 14.29 | 27.16 |
| 1997 | 23.00 | 12.18 | 23.14 |
| 1998 | 22.00 | 11.65 | 22.13 |
| 1999 | 30.00 | 15.88 | 30.18 |
| 2000 | 52.40 | 39.00 | 74.10 |
| 2001 | 72.40 | 34.00 | 64.60 |
| 2002 | 125.00 | 33.00 | 62.70 |
| 2003 | 177.74 | 50.00 | 95.00 |
| 2004 | 236.40 | 64.00 | 121.60 |
| 2005 | 316.00 | 85.00 | 161.50 |
| 2006 | 530.00 | 100.00 | 190.00 |
| 2007 | 720.60 | 107.00 | 203.30 |
| 2008 | 694.30 | 130.00 | 247.00 |
| 2009 | 1143.07 | 162.50 | 308.75 |
| sum | 4347.31 | 952.42 | 1809.59 |

The data of stainless steel production was obtained through Almanac of China Hardware Industry. And the ferrochromium production data before 2001 was calculated by the assumption that0.45 t ferrochromium could produce 1t stainless steel. The data of ferrochromium production from 2002 to 2006 were obtained from the website <http://www.indexmundi.com/minerals/?country=cn&product=ferrochromium&graph=production>, and the data of 2001 and 2007-2009 came from report[26]and online data.

**Table S4.The estimated discharge into water from nonferrous mining industry**

| Year | Domestically mined chrome(10^4^t) | Import chrome(10^4^t) | Estimated emission(t) |
| --- | --- | --- | --- |
| 1990 | 10.28 | 80.68 | 22.93 |
| 1991 | 10.70 | 54.46 | 17.52 |
| 1992 | 10.22 | 90.10 | 24.92 |
| 1993 | 15.42 | 61.88 | 21.68 |
| 1994 | 5.69 | 65.10 | 17.09 |
| 1995 | 19.80 | 138.10 | 40.44 |
| 1996 | 12.84 | 76.44 | 23.41 |
| 1997 | 17.68 | 89.40 | 28.82 |
| 1998 | 20.53 | 71.47 | 26.51 |
| 1999 | 22.05 | 78.95 | 28.95 |
| 2000 | 20.80 | 111.30 | 35.22 |
| 2001 | 18.19 | 109.01 | 33.31 |
| 2002 | 13.00 | 114.00 | 31.57 |
| 2003 | 10.00 | 178.00 | 43.70 |
| 2004 | 23.33 | 217.00 | 59.33 |
| 2005 | 20.00 | 302.00 | 75.79 |
| 2006 | 21.86 | 432.43 | 104.84 |
| 2007 | 23.46 | 608.91 | 143.65 |
| 2008 | 21.95 | 401.16 | 98.17 |
| 2009 | 19.70 | 616.20 | 143.18 |

The data of ore mining before 2003 were cited from Zhang [27], and other years’ data was surveyed online, <http://www.baiinfo.com>. The emission co-efficient for the two categories is 54.3 g/t and 21.5 g/t , respectively, which was taken from the Manual for Coefficients of Pollutant Generation and Discharge in Industrial Pollution Sources of China.

**Table S5 The estimated discharge into water from leather tanning industry**

| Year | Light leather(10^5^m^2^) | Leather shoes(10^8^ pairs) | ^a^Emission of light leather(t) | ^b^Emission of heavy leather(t) | Total(t) |
| --- | --- | --- | --- | --- | --- |
| 1990 | 13578.11 | 4.38 | 27.40 | 10.60 | 38.00 |
| 1991 | 14668.65 | 5.36 | 29.60 | 12.98 | 42.59 |
| 1992 | 15192.32 | 7.71 | 30.66 | 18.66 | 49.33 |
| 1993 | 19723.07 | 9.15 | 39.81 | 22.16 | 61.97 |
| 1994 | 19389.14 | 15.54 | 39.13 | 37.65 | 76.78 |
| 1995 | 25888.39 | 22.60 | 52.25 | 54.73 | 106.98 |
| 1996 | 23515.01 | 24.30 | 47.46 | 58.85 | 106.31 |
| 1997 | 23991.52 | 24.70 | 48.42 | 59.82 | 108.24 |
| 1998 | 27108.00 | 25.10 | 54.71 | 60.79 | 115.50 |
| 1999 | 31680.00 | 21.90 | 63.94 | 53.04 | 116.98 |
| 2000 | 38126.00 | 23.70 | 76.95 | 57.40 | 134.35 |
| 2001 | 45900.00 | 21.00 | 92.64 | 50.86 | 143.50 |
| 2002 | 50400.00 | 23.80 | 101.72 | 57.64 | 159.36 |
| 2003 | 53000.00 | 26.70 | 106.97 | 64.66 | 171.63 |
| 2004 | 56000.00 | 28.30 | 113.02 | 68.54 | 181.56 |
| 2005 | 60588.00 | 29.70 | 122.28 | 71.93 | 194.21 |
| 2006 | 72419.00 | 30.77 | 146.16 | 74.52 | 220.68 |
| 2007 | 68394.00 | 33.64 | 138.04 | 81.47 | 219.51 |
| 2008 | 64204.00 | 33.15 | 129.58 | 80.29 | 209.86 |
| 2009 | 69205.00 | 35.46 | 139.67 | 85.88 | 225.55 |

Based on the Manual for Coefficients of Pollutant Generation and Discharge in Industrial Pollution Sources, the average emission factor of leather is 30g/t. The production data of light leather and heavy leather came from China Light Industry Yearbook from 1991-2010.

a, b: For leather production, we use following conversion system to calculate the emission: a single piece of leather equivalent to 25 kg of leather or 40 square feet of finished leather.

**Table S6 Industrial output values of sectors and discount rate of China from 1990 to 2009(in 10^8^ RMB)**

| Year | NMSP | FMI | MI | CMI | NMMD | OI | Total | Interest rate(%) | Discount rate |
| --- | --- | --- | --- | --- | --- | --- | --- | --- | --- |
| 1990 | 509.46 | 522.57 | 199.11 | 1492.01 | 103.11 | 16062.07 | 18689.22 | 9.36 | 1.00 |
| 1991 | 573.55 | 615.23 | 253.35 | 1625.07 | 113.45 | 19161.38 | 22088.68 | 7.56 | 1.08 |
| 1992 | 709.11 | 800.43 | 325.20 | 1911.16 | 125.63 | 24177.88 | 27724.21 | 7.56 | 1.16 |
| 1993 | 974.45 | 1302.05 | 570.73 | 2377.00 | 191.26 | 34848.24 | 39693.00 | 10.08 | 1.27 |
| 1994 | 1202.36 | 1707.99 | 843.41 | 3165.33 | 227.54 | 45049.81 | 51353.03 | 10.08 | 1.40 |
| 1995 | 1372.29 | 1650.72 | 974.41 | 3819.79 | 111.93 | 47992.13 | 54946.86 | 10.08 | 1.54 |
| 1996 | 1424.55 | 1943.78 | 1112.20 | 4471.36 | 145.78 | 54754.69 | 62740.16 | 8.32 | 1.67 |
| 1997 | 1470.00 | 2078.10 | 1186.36 | 4722.37 | 389.56 | 59692.65 | 68352.68 | 5.67 | 1.77 |
| 1998 | 1628.73 | 2150.68 | 1191.93 | 4627.83 | 339.02 | 58990.88 | 67737.14 | 4.59 | 1.85 |
| 1999 | 1793.14 | 2215.09 | 1197.93 | 4924.78 | 361.52 | 63412.51 | 72707.04 | 2.25 | 1.89 |
| 2000 | 2180.23 | 2539.76 | 1345.17 | 5749.02 | 405.36 | 74799.29 | 85673.66 | 2.25 | 1.93 |
| 2001 | 2369.17 | 2852.27 | 1572.63 | 6303.66 | 419.15 | 83504.73 | 95448.98 | 2.25 | 1.98 |
| 2002 | 2599.98 | 3294.38 | 1801.46 | 7220.05 | 463.90 | 97198.17 | 110776.48 | 1.98 | 2.01 |
| 2003 | 3564.07 | 3857.40 | 2274.05 | 9244.86 | 573.28 | 125031.61 | 142271.22 | 1.98 | 2.05 |
| 2004 | 5009.09 | 4013.60 | 1669.80 | 12118.60 | 710.39 | 173410.01 | 195261.69 | 2.25 | 2.10 |
| 2005 | 6244.08 | 6359.66 | 3133.23 | 14027.74 | 913.53 | 194770.92 | 222315.93 | 2.25 | 2.15 |
| 2006 | 12936.48 | 8529.47 | 4150.04 | 20448.69 | 1671.73 | 273002.59 | 316588.96 | 2.52 | 2.20 |
| 2007 | 18031.88 | 11447.08 | 5153.49 | 26798.80 | 2288.75 | 346610.62 | 405177.13 | 3.46 | 2.28 |
| 2008 | 20948.74 | 15029.61 | 5871.43 | 33955.07 | 2727.84 | 434786.99 | 507448.25 | 3.06 | 2.35 |
| 2009 | 20567.21 | 16082.95 | 6425.57 | 36908.63 | 2814.67 | 471937.96 | 548311.42 | 3.06 | 2.42 |

NMSP: Nonferrous smelting and pressing; FMI: Fabricated metal industry; LI: Leather industry; CMI: Chemical manufacturing industry; NMMD: Nonferrous mining and dressing; OI: Other industries

The industrial output value came from China Statistical Yearbook (1991-2010).

All interest rate of one year came from Bank of China. When one year has different interest rate, we assume the average of them to be interest rate of that year. The discount rate is calculated by the following equation.

$$D_{i+1}=D_{i}\times(1+R_{i+1})$$

D_i+1_ is the discount rate of (i+1) year; R_i+1_ is the interest rate of (i+1) year; D_i_ is the discount rate of i year.

**Table S7.The estimated atmospheric chromium emissions in China, 1990-2009(in tons)**

| Year | CC | WI | CP | FP | OC | ISP | Sum |
| --- | --- | --- | --- | --- | --- | --- | --- |
| 1990 | 2702.25 | 0.00 | 209.71 | 24.14 | 1143.26 | 408.87 | 4488.23 |
| 1991 | 2787.34 | 0.00 | 252.61 | 26.15 | 1312.25 | 439.60 | 4817.95 |
| 1992 | 2948.83 | 0.00 | 308.22 | 25.15 | 1452.66 | 498.31 | 5233.17 |
| 1993 | 3117.54 | 0.00 | 367.88 | 32.09 | 1685.25 | 559.24 | 5761.99 |
| 1994 | 3412.01 | 0.00 | 421.18 | 32.69 | 1646.03 | 594.48 | 6106.39 |
| 1995 | 3771.47 | 0.00 | 475.61 | 38.22 | 1701.93 | 623.61 | 6610.85 |
| 1996 | 3762.99 | 0.00 | 491.19 | 27.16 | 1781.62 | 651.58 | 6714.54 |
| 1997 | 3720.16 | 0.00 | 511.74 | 23.14 | 1848.53 | 700.53 | 6804.09 |
| 1998 | 3361.61 | 0.00 | 536.00 | 22.13 | 1982.99 | 735.22 | 6637.96 |
| 1999 | 2451.81 | 0.00 | 573.00 | 30.18 | 2139.66 | 785.44 | 5980.08 |
| 2000 | 2279.15 | 0.00 | 597.00 | 74.10 | 2342.74 | 815.33 | 6108.33 |
| 2001 | 2695.83 | 0.00 | 661.04 | 64.60 | 2512.57 | 964.29 | 6898.32 |
| 2002 | 3420.94 | 0.00 | 725.00 | 62.70 | 2664.81 | 1122.41 | 7995.86 |
| 2003 | 4305.71 | 4.07 | 862.08 | 95.00 | 3081.45 | 1380.78 | 9729.08 |
| 2004 | 5565.94 | 4.94 | 933.69 | 121.60 | 3614.28 | 1964.94 | 12205.39 |
| 2005 | 6578.79 | 8.70 | 1068.85 | 161.50 | 4590.52 | 2380.21 | 14788.57 |
| 2006 | 7275.51 | 12.51 | 1236.76 | 190.00 | 5072.65 | 2625.23 | 16412.68 |
| 2007 | 7900.18 | 15.78 | 1361.17 | 203.30 | 5629.58 | 3297.78 | 18407.80 |
| 2008 | 8393.14 | 17.27 | 1388.38 | 247.00 | 5972.40 | 3084.50 | 19102.69 |
| 2009 | 8880.74 | 22.24 | 1643.98 | 308.75 | 6364.69 | 3846.34 | 21066.73 |
| sum | 89331.93 | 85.52 | 14625.09 | 1809.59 | 58539.89 | 27478.70 | 191870.72 |

* CC: Coal Combustion; WI: Waste Incineration; CP: Cement Production; FP: Ferrochromium production; OC: Oil Combustion; ISP: Iron and Steel Production.

**Table S8. The estimated atmospheric chromium emissions by region in China, 2005-2009(in tons)**

| Region | 2005 | 2006 | 2007 | 2008 | 2009 | Sum |
| --- | --- | --- | --- | --- | --- | --- |
| **Northern Region** | **3239.17** | **3592.85** | **4098.61** | **4167.57** | **4630.51** | **19728.70** |
| Beijing | 307.47 | 341.92 | 374.67 | 370.34 | 391.89 | 1786.29 |
| Tianjin | 265.23 | 291.57 | 329.30 | 336.96 | 392.02 | 1615.07 |
| Hebei | 1326.29 | 1461.58 | 1747.32 | 1724.06 | 2001.06 | 8260.31 |
| Shanxi | 918.42 | 1012.01 | 1076.21 | 1076.21 | 1110.08 | 5192.94 |
| Inner Mongolia | 421.76 | 485.77 | 571.12 | 660.00 | 735.44 | 2874.09 |
| **Northeastern Region** | **1523.56** | **1676.81** | **1891.63** | **1835.89** | **2057.49** | **8985.37** |
| Liaoning | 830.49 | 922.81 | 1052.16 | 1041.87 | 1169.19 | 5016.52 |
| Jilin | 288.25 | 326.30 | 376.32 | 341.96 | 386.83 | 1719.67 |
| Heilongjiang | 404.81 | 427.69 | 463.15 | 452.06 | 501.47 | 2249.17 |
| **Eastern Region** | **4754.75** | **5249.76** | **5841.26** | **6014.46** | **6477.45** | **28337.68** |
| Shanghai | 497.03 | 517.81 | 573.09 | 583.00 | 634.65 | 2805.59 |
| Jiangsu | 951.87 | 1065.36 | 1201.17 | 1264.63 | 1377.76 | 5860.79 |
| Zhejiang | 735.77 | 791.86 | 857.87 | 885.81 | 931.01 | 4202.32 |
| Anhui | 458.71 | 496.23 | 572.51 | 621.00 | 682.33 | 2830.78 |
| Fujian | 365.66 | 400.25 | 478.04 | 480.24 | 511.33 | 2235.53 |
| Jiangxi | 354.22 | 386.72 | 421.57 | 422.44 | 468.89 | 2053.84 |
| Shandong | 1391.48 | 1591.53 | 1737.01 | 1757.34 | 1871.47 | 8348.84 |
| **Central and Southern** | **3500.74** | **3879.74** | **4325.74** | **4513.88** | **4900.11** | **21120.20** |
| Henan | 742.39 | 867.62 | 985.42 | 1028.99 | 1091.06 | 4715.48 |
| Hubei | 646.72 | 707.32 | 784.45 | 866.62 | 949.97 | 3955.09 |
| Hunan | 592.49 | 641.80 | 718.16 | 683.80 | 763.86 | 3400.11 |
| Guangdong | 1040.56 | 1127.72 | 1234.09 | 1304.59 | 1375.49 | 6082.46 |
| Guangxi | 422.85 | 473.39 | 537.31 | 549.24 | 623.71 | 2606.51 |
| Hainan | 55.72 | 61.88 | 66.31 | 80.64 | 96.01 | 360.56 |
| **Southwestern Region** | **1749.46** | **1935.45** | **2138.64** | **2349.86** | **2676.52** | **10849.93** |
| Chongqing | 238.98 | 239.57 | 266.58 | 327.88 | 357.87 | 1430.89 |
| Sichuan | 540.84 | 616.89 | 729.87 | 825.34 | 994.71 | 3707.66 |
| Guizhou | 292.81 | 345.90 | 375.64 | 357.14 | 392.90 | 1764.39 |
| Yunnan | 675.54 | 731.41 | 764.94 | 837.84 | 929.17 | 3938.91 |
| Tibet | 1.28 | 1.67 | 1.60 | 1.67 | 1.88 | 8.09 |
| **Northwestern Region** | **862.35** | **992.30** | **1134.13** | **1225.74** | **1358.29** | **5572.81** |
| Qinghai | 32.96 | 42.16 | 56.59 | 72.61 | 80.07 | 284.39 |
| Ningxia | 112.38 | 116.64 | 137.37 | 142.69 | 163.87 | 672.96 |
| Gansu | 180.57 | 189.40 | 204.21 | 200.88 | 216.14 | 991.20 |
| Xinjiang | 203.64 | 239.92 | 269.16 | 274.65 | 312.85 | 1300.22 |
| Shaanxi | 332.80 | 404.17 | 466.79 | 534.91 | 585.36 | 2324.04 |
| Sum | 15630.02 | 17326.91 | 19430.00 | 20107.40 | 22100.36 | 94594.70 |

**Table S9.The estimated chromium emissions to water in China, 1990-2009(in tons)**

| Year | NMSP | FMI | MI | CMI | NMMD | OI | Total |
| --- | --- | --- | --- | --- | --- | --- | --- |
| 1990 | 0.08 | 111.39 | 38.00 | 1.50 | 22.93 | 7.00 | 180.91 |
| 1991 | 0.08 | 121.43 | 42.59 | 1.51 | 17.52 | 7.67 | 190.80 |
| 1992 | 0.10 | 147.09 | 49.33 | 1.66 | 24.92 | 8.96 | 232.05 |
| 1993 | 0.13 | 239.27 | 61.97 | 2.06 | 21.68 | 12.82 | 337.93 |
| 1994 | 0.13 | 260.06 | 76.78 | 2.27 | 17.09 | 11.83 | 368.17 |
| 1995 | 0.14 | 228.49 | 106.98 | 2.49 | 40.44 | 11.44 | 389.99 |
| 1996 | 0.13 | 248.11 | 106.31 | 2.69 | 23.41 | 12.04 | 392.69 |
| 1997 | 0.13 | 250.27 | 108.24 | 2.68 | 28.82 | 12.39 | 402.54 |
| 1998 | 0.14 | 247.81 | 115.50 | 2.52 | 26.51 | 11.71 | 404.19 |
| 1999 | 0.15 | 249.83 | 116.98 | 2.62 | 28.95 | 12.34 | 410.86 |
| 2000 | 0.18 | 280.51 | 134.35 | 3.00 | 35.22 | 14.26 | 467.52 |
| 2001 | 0.19 | 307.08 | 143.50 | 3.20 | 33.31 | 15.51 | 502.78 |
| 2002 | 0.20 | 349.38 | 159.36 | 3.61 | 31.57 | 17.79 | 561.91 |
| 2003 | 0.27 | 401.11 | 171.63 | 4.54 | 43.70 | 22.44 | 643.69 |
| 2004 | 0.37 | 407.41 | 181.56 | 5.80 | 59.33 | 30.65 | 685.12 |
| 2005 | 0.44 | 616.21 | 194.21 | 6.41 | 75.79 | 32.65 | 925.71 |
| 2006 | 0.91 | 826.45 | 220.68 | 9.35 | 104.84 | 45.80 | 1208.04 |
| 2007 | 1.23 | 1070.24 | 219.51 | 11.82 | 143.65 | 56.13 | 1502.58 |
| 2008 | 1.35 | 1323.89 | 209.86 | 14.11 | 98.17 | 66.43 | 1713.81 |
| 2009 | 1.32 | 1416.67 | 225.55 | 15.34 | 143.18 | 72.10 | 1874.16 |

NMSP: Nonferrous smelting and pressing; FMI: Fabricated metal industry; LI: Leather industry; CMI: Chemical manufacturing industry; NMMD: Nonferrous mining and dressing industry; OI: Other industries

**Table S10. Emission factors of different types of oil combustion.**

| Categories | Emission factor（g/t） | Literature cited |
| --- | --- | --- |
| Diesel^a^ | 30.4083g/t | [28] |
| Kerosene^a^ | 30.3622g/t | [28] |
| Gasoline | 16g/t | [28] |

a: The emission factor for diesel and kerosene is 1.4 g/GJ. The calorific values are 46.04 MJ/kg and 43.11 MJ/kg, respectively.

b: The emission factor for fuel oil is 8.45*10^-4^ lb/10^3^ gal. To convert from lb/10^3^ gal to g/t, multiply by 0.12

**Table S11. Emission factors of ferrochromium production and related industries**

| Categories | Emission factor(g/t) | Literature cited |
| --- | --- | --- |
| ferrochromium production | 190 | [9] |
| Cement production | 1 | [2] |
| Iron and steel production |  |  |
| Iron production | 2.3 | [28] |
| Steel production | 4.5 | [28] |
| Waste incarnation | 1.1 | [2] |

# Figures

**Figure S1. The temporal trend of emission of chromium from coal combustion**

*For four sectors of coal combustion have drastic difference of the industry sector and power plant sector based on the left vertical axis, and others and residential sector based on right vertical axis.

Among all of the coal consuming sectors, the chromium emissions from the power sector are increasing the fastest, with an average 7.91% annual increase, reaching 1407 t in 2009. However, the main contributor to coal combustion is the industry sector, which remained nearly constant throughout study period, representing over 80% of coal combustion. Another highlighted feature is that the emissions of chromium from the industry have increased substantially since 2005. The rapid expansion of energy-intensive manufacturing industries, such as steel and cement production, and coal consumption by industrial sector after the recovery of the economic decline may explain this rapid growth. The growth rate of power plants is lower compared with that of the industrial sector, and negative growth was observed in 2004 and 2008. This may due to the improvement of PM and SO_2_ control devices in coal-fired power plants[29].

Emission from the residential sector and other sectors declined at the beginning of the study period and then increased after 2003. This fluctuation may be a co-effect of the substitution of coal with cleaner fuels, such as natural gas, and the gradual increase of coal consumption in recent years.

**Figure S2. The temporal trend of emission of chromium from oil combustion**

**References:**

1. Tian HZ, Cheng K, Wang Y, Zhao D, Lu L, et al. (2012) Temporal and spatial variation characteristics of atmospheric emissions of Cd, Cr, and Pb from coal in China. Atmospheric Environment 50: 157-163.

2. Jozef MP, Elisabeth GP (2001) An assessment of global and regional emissions of trace metals to the atmosphere from anthropogenic sources worldwide. Environmental Reviews 9: 269.

3. Tang X, Zhao J, Huang W (2002) Nine Metal Elements in Coal of China. Coal Geology of China 14: 43-54.

4. Xu M, Yan R, Zheng C, Qiao Y, Han J, et al. (2004) Status of trace element emission in a coal combustion process: a review. Fuel Processing Technology 85: 215-237.

5. Tian HZ, Cheng K, Wang Y, Zhao D, Chai FH, et al. (2011) Quantitative Assessment of Variability and Uncertainty of Hazardous Trace Element (Cd, Cr, and Pb) Contents in Chinese Coals by Using Bootstrap Simulation. Journal of the Air & Waste Management Association 61: 755-763.

6. Tian HZ, Wang Y, Xue ZG, Cheng K, Qu YP, et al. (2010) Trend and characteristics of atmospheric emissions of Hg, As, and Se from coal combustion in China, 1980-2007. Atmospheric Chemistry and Physics 10: 11905-11919.

7. Li Q, Cheng H, Zhou T, Lin C, Guo S (2012) The estimated atmospheric lead emissions in China, 1990-2009. Atmospheric Environment 60: 1-8.

8. EPA USEP (2001) Compilation of Air Pollutant Emission Factors, AP-42. <http://www.epa.gov/ttnchie1/ap42/>

9. Shular J (1989) Locating and Estimating Air Emissions from Sources of Chromium: Supplement: US Environmental Protection Agency, Office of Air Quality Planning and Standards.

10. Bergbäck B, Anderberg S, Lohm U (1989) A reconstruction of emission, flow and accumulation of chromium in sweden 1920–1980. Water, Air, & Soil Pollution 48: 391-407.

11. Chen J, Wang S, Lin W, Zhang M, Zhao J (2005) Development and resource status of China chrome industry in the 21th century. Ferro-alloys 2: 11.

12. Wu Y, Wang S, Streets DG, Hao J, Chan M, et al. (2006) Trends in Anthropogenic Mercury Emissions in China from 1995 to 2003. Environmental Science & Technology 40: 5312-5318.

13. Worrell E, Price L, Martin N, Hendriks C, Meida LO (2001) Carbon dioxide emissions from the global cement industry 1. Annual Review of Energy and the Environment 26: 303-329.

14. Owlad M, Aroua MK, Daud WAW, Baroutian S (2009) Removal of hexavalent chromium-contaminated water and wastewater: a review. Water, Air & Soil Pollution 200: 59-77.

15. Deng S, Bai R (2004) Removal of trivalent and hexavalent chromium with aminated polyacrylonitrile fibers: performance and mechanisms. Water research 38: 2424-2432.

16. Nodelman IG, Pisupati SV, Miller SF, Scaroni AW (2000) Partitioning behavior of trace elements during pilot-scale combustion of pulverized coal and coal–water slurry fuel. Journal of Hazardous Materials 74: 47-59.

17. Huang Y, Jin B, Zhong Z, Xiao R, Tang Z, et al. (2004) Trace elements (Mn, Cr, Pb, Se, Zn, Cd and Hg) in emissions from a pulverized coal boiler. Fuel Processing Technology 86: 23-32.

18. Wang QC, Kang SL, Chen C, Wang ZG, Zou ST (1996) Study on the contents and distribution laws of trace elements in coal in northeast China and eastern Inner Mongolia. Environmental Chemistry 15: 27-35.

19. Zhang J, Han C, Xu Y (2003) The release of the hazardous elements from coal in the initial stage of combustion process. Fuel Processing Technology 84: 121-133.

20. Åmand L, Leckner B, Eskilsson D, Tullin C (2006) Deposits on heat transfer tubes during co-combustion of biofuels and sewage sludge. Fuel 85: 1313-1322.

21. Ma J (2008) Study on the transformation of hazardous trace elements during the coal-coking: Wuhan Technology University.

22. Helble JJ, Mojtahedi W, Lyyränen J, Jokiniemi J, Kauppinen E (1996) Trace element partitioning during coal gasification. Fuel 75: 931-939.

23. Meij R, Te Winkel H (2007) The emissions of heavy metals and persistent organic pollutants from modern coal-fired power stations. Atmospheric Environment 41: 9262-9272.

24. Nyberg CM, Thompson JS, Zhuang Y, Pavlish JH, Brickett L, et al. (2009) Fate of trace element haps when applying mercury control technologies. Fuel Processing Technology 90: 1348-1353.

25. Yi H, Hao J, Duan L, Tang X, Ning P, et al. (2008) Fine particle and trace element emissions from an anthracite coal-fired power plant equipped with a bag-house in China. Fuel 87: 2050-2057.

26. Liu G (2009) Analysis of chromium alloys production and operation in China from 2008 to 2009. China Steel Focus: 14-18.

27. Zhang F, Gao P, Chen J (2005) The further supply and requirement situation of ore for China. Contribution to Geology and Mineral Resource Research 20: 215-217.

28. European Environmental Agency (EEA) (2009) EEA air pollutant emission inventory guidebook 2009. <http://www.eea.europa.eu/publications/emep-eea-emission-inventory-guidebook-2009>

29. Zhao Y, Wang S, Duan L, Lei Y, Cao P, et al. (2008) Primary air pollutant emissions of coal-fired power plants in China: Current status and future prediction. Atmospheric Environment 42: 8442-8452.
